# Comprehensive comparison of *Yarrowia lipolytica* and *Pichia pastoris* for production of *Candida antarctica* lipase B

**DOI:** 10.1038/s41598-020-58683-3

**Published:** 2020-02-03

**Authors:** Chrispian W. Theron, Marie Vandermies, Samuel Telek, Sebastien Steels, Patrick Fickers

**Affiliations:** Microbial Processes and Interactions, TERRA Teaching and Research Centre, University of Liège - Gembloux AgroBio Tech, Avenue de la Faculté, 2. B-, 5030 Gembloux, Belgium

**Keywords:** Expression systems, Industrial microbiology

## Abstract

The large-scale production of recombinant proteins (rProt) is becoming increasingly economically important. Among the different hosts used for rProt production, yeasts are gaining popularity. The so-called non-conventional yeasts, such as the methylotrophic *Pichia pastoris* and the dimorphic *Yarrowia lipolytica*, are popular choices due to their favorable characteristics and well-established expression systems. Nevertheless, a direct comparison of the two systems for rProt production and secretion was lacking. This study therefore aimed to directly compare *Y. lipolytica* and *P. pastoris* for the production and secretion of lipase CalB in bioreactor. *Y. lipolytica* produced more than double the biomass and more than 5-fold higher extracellular lipase than *P. pastoris*. Furthermore, maximal CalB production levels were reached by *Y. lipolytica* in half the cultivation time required for maximal production by *P. pastoris*. Conversely, *P. pastoris* was found to express 7-fold higher levels of CalB mRNA. Secreted enhanced green fluorescent protein –in isolation and fused to CalB– and protease inhibitor MG-132 were used in *P. pastoris* to further investigate the reasons behind such discrepancy. The most likely explanation was ultimately found to be protein degradation by endoplasmic reticulum-associated protein degradation preceding successful secretion. This study highlighted the multifaceted nature of rProt production, prompting a global outlook for selection of rProt production systems.

## Introduction

The production of recombinant proteins (rProt) at industrial scale is of increasing economic importance. Among the different microbial chassis that have been developed for that purpose, yeasts are emerging as the preferred option for the production of recombinant enzymes and therapeutic proteins. The main advantage of yeasts over bacterial systems such as *Escherichia coli* is the possibility to obtain post-translational modified proteins in the culture supernatant at a gram per liter scale. Historically, *Saccharomyces cerevisiae* has been used as the reference eukaryotic chassis, however it suffers several drawbacks such as low protein productivity, overflow metabolism and hyperglycosylation of rProt. Moreover, it is less metabolically adapted to catabolize raw carbon and nitrogen sources, which are nowadays increasingly considered as feedstocks in bioprocesses, with the intention of reducing the process costs. Currently, non-conventional yeasts such as *Pichia pastoris* and *Yarrowia lipolytica* are considered as realistic alternatives to *S. cerevisiae* for rProt synthesis. Both species combine the advantages of growth at high cell density and production and secretion of rProt at high yields, with low nutritional requirements, thus allowing growth on raw materials or industrial by-products^1,2^.

In most cases, the processes developed for rProt production are two-step systems involving a first phase of biomass generation under repressive or non-inducing conditions, followed by an induction phase during which rProt are synthetized and secreted into the culture medium. Such a strategy has many advantages over a continuous system, e. g. a lower global cellular metabolic load and a reduced risk of alteration of rProt in the harsh environment of culture medium.

*Y. lipolytica* is a dimorphic yeast isolated from protein and lipid-rich environments (reviewed by Nicaud^3^). This species is thus equipped with efficient and specific catabolic pathways for proteins and lipids^4^. In protein-rich media, alkaline extracellular protease can be secreted up to 1–2 g.L^−1^, while in lipid-rich medium, lipases, such as Lip2p, are secreted at high yields^5,6^. These peculiar metabolic traits have been exploited to develop molecular tools for rProt synthesis and secretion^7^. When combined with efficient bioreactor process strategies, these tools have been successfully used for the production of a large number of rProt^8,9^. We have recently developed a novel set of expression vectors based on the promoter of *EYK1* gene encoding erythrulose kinase^10,11^. Compared to previously available inducible systems like *pPOX2* and *pLIP2*, this promoter does not depend on the utilization of hydrophobic inducers such as oleic acid^12^. Instead, it is strongly induced in the presence of erythritol, which can be used both as a carbon source and as an inducer, and it is repressed in the presence of glucose or glycerol. When combined with an *eyk1*Δ recipient strain, erythritol could also be used as a free inducer in combination with another carbon source (usually glycerol) for energy generation. Furthermore, the identification of the *EYK1* promoter regulatory elements led the development of hybrid promoters allowing subsequent fine-tuning of the gene expression level^10^.

The non-conventional yeast *P. pastoris* is a well-established yeast system for rProt production, as currently more than 1000 rProt have been produced using this yeast^1,13^. This yeast is well known for its ability to grow on methanol as sole carbon source. It relies on a specific catabolic pathway starting in peroxisomes and based on the high expression level of different genes, including *AOX1* encoding methanol oxidase^14^. This specific physiological trait has been the starting point for the development of efficient expression vectors, notably involving the *AOX1* promoter (p*AOX1*). In addition, the phenotype of the producing strain in regards to methanol catabolic efficiency (Mut+, MutS, Mut−)^15^ affects the production of rProt. MutS strains have often been demonstrated to produce higher rProt yields than Mut+ strains^15–17^, which comes with the added advantage of requiring lower levels of methanol as inducer. The latter is an important consideration since the use of methanol on large-scale presents drawbacks such as toxicity and flammability, high oxygen consumption and high heat release. Another solution to minimize methanol addition involves different co-feeding processes that have been successfully developed, where part of the methanol used as inducer and energy source has been replaced by glycerol^15^ or sorbitol^18^.

Although many examples exist in the literature demonstrating the performance of both *P. pastoris* and *Y. lipolytica*, none of them reported a direct comparison of the same secreted rProt produced at bioreactor scale. Here, we selected a protein of industrial interest, the lipase B from *Candida antarctica* (CalB), to compare the production and secretion abilities of both cell factories. Expression systems that have previously led to promising rProt production in both strains in our prior research were selected, and comparisons were made at both the gene expression and final protein levels. Furthermore, potential reasons behind the observed differences were deciphered.

## Results and Discussion

### CalB sequence analysis and cloning

The DNA fragment containing the coding sequence of lipase CalB and its pro-region was codon optimized and synthesized during previous work^11^ (for pro-CalB coding sequence, see Supplementary Fig. 1). It was cloned in expression vectors specific for *Y. lipolytica* (JMP4266, promoter p*EYK1-A3B*, *URA3* marker) and *P. pastoris* (pIB4, promoter p*AOX1*, *HIS4* marker). The nucleotide sequence of pro-CalB was analyzed for the presence of less abundant codons (i.e., less than 0.2 in frequency) and rare codons (i.e., less than 0.1 in frequency). For *Y. lipolytica*, only two codons TCG coding for Ser77 and Ser229 were found with a frequency of 0.16 (for a graphical view of these results, see Supplementary Fig. 1). No rare codons were identified. For *P. pastoris*, neither less abundant codons nor rare codons could be identified. This suggests that protein translation should not be a limiting factor of CalB synthesis in either recipient strain.

In *Y. lipolytica*, the pro-CalB gene was cloned under the control of the hybrid *pEYK1–3AB* promoter, and signal sequence of *LIP2* encoding extracellular lipase was used for secretion^19^. The resulting construct was integrated at the zeta docking platform of strain JMY7126 to yield prototroph strain RIY368.

For expression in *P. pastoris*, the pro-CalB DNA sequence or the mature sequence (i.e., excluding the pro-region) were fused with α-mating factor from *S. cerevisiae* and cloned under the control of p*AOX1* promoter. The resulting constructs were integrated into the genome of a MutS strain (i.e., strain RIY282) at the *HIS4* locus, to yield RIY311 and RIY314 respectively. The mature CalB-encoding sequence was also fused to the enhanced green fluorescent protein (EGFP) in strain RIY309.

After transformation, three positive transformants for each construct of each yeast were tested for extracellular CalB lipase activity. No significant difference in lipase activity (less than 15%) could be observed between transformants, demonstrating thus that only a single expression cassette was integrated in the genome and inter-clone variation was negligible.

Due to differences in genomic architecture, standardization of specific integration loci between species in unfeasible. However, studies in both *P. pastoris*^20^ and *Y. lipolytica*^21^ demonstrated reasonable expression leeway between different integration loci in both strains. Nevertheless, both of the integration targets used in this study, the *LEU2* locus (where the zeta element docking platform has been introduced) in *Y. lipolytica* and the *HIS4* locus in *P. pastoris*, are among the most commonly used integration targets for the respective species^22^.

### Cell growth and carbon uptake

**Figure 1:**
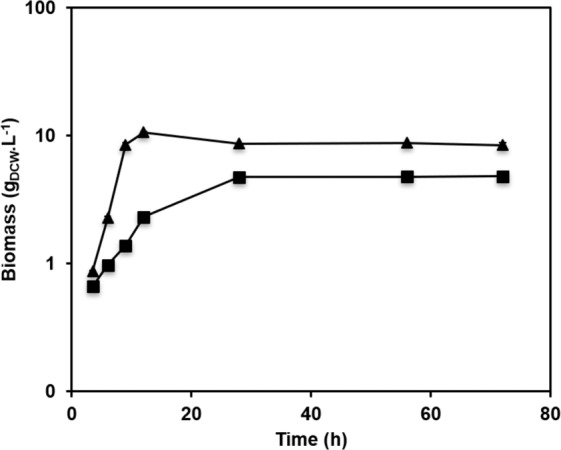
Cell growth of *Y. lipolytica* RIY368 (triangles) and *P. pastoris* RIY311 (squares) during culture in bioreactor. Cells were grown for 72 h in YSPGE and in YSPSM medium, respectively. Displayed values correspond to the means of independent duplicate experiments. Standard deviations were less than 4.9% for *Y. lipolytica* and 7.4% for *P. pastoris*.

As a first characterization, the growth kinetics of both yeast species were compared during bioreactor cultures in a medium previously demonstrated efficient for extracellular protein production^16^. For *Y. lipolytica*, the buffered rich medium was supplemented with glycerol as a carbon source, and erythritol as the inducer (YSPGE medium). Indeed, glycerol has been previously demonstrated to be a more efficient carbon source than glucose^23^, and it is available at low cost as a by-product of the biodiesel industry. For *P. pastoris*, the buffered rich medium was supplemented with a mixture of sorbitol and methanol (YSPSM medium). The carbon source content was calculated to obtain the same molar concentration of metabolizable carbon (0.6 Cmol.L^−1^) for both species. As shown in Fig. 1 and Table 1, the kinetics of growth were somewhat different for the two yeast species. *Y. lipolytica* strain RIY368 (p*EYK1–3AB*-pro-CalB) grew faster, with specific growth rate of 0.31 h^−1^ during the exponential growth phase (i.e., between 6 and 12 h of culture), and reached a maximal biomass of 10.6 g_DCW_.L^−1^ after 12 h. The specific growth rate obtained here is in the same range that the one previously determined on glycerol (0.31 h^−1^)^23^. Glycerol was consumed at a rate of 0.05 g.g_DCW_^−1^.h^−1^ within 12 h. *P. pastoris* strain RIY311 (p*AOX1*-αMF-pro-CalB) grew somewhat slower, with a specific growth rate of 0.27 h^−1^ during the exponential growth phase, and reached a maximum biomass of 4.8 g_DCW_.L^−1^ by the end of the culture. As previously highlighted, *P. pastoris* is able to co-metabolize sorbitol and methanol^18^. Here, it occurred with a higher rate for sorbitol (0.07 g.g_DCW_^−1^.h^−1^) as compared to methanol (0.02 g.g_DCW_^−1^.h^−1^). It is important to remember that the strain used in this study was MutS and hence methanol metabolism is slower than in wild-type strains. *Y. lipolytica* RIY368 also used carbon sources more efficiently than *P. pastoris* RIY311, as demonstrated by the values of cell yield coefficients (16.8 versus 8 g_DCW_.molC^−1^).

**Table 1:** Dynamics of growth and carbon uptake rate during cultures in bioreactor. Displayed values correspond to the means and standard deviations of independent duplicate experiments.

	*Y. lipolytica* RIY368	*P. pastoris* RIY311
Growth rate (h^−1^)	0.31 ± 0.06	0.27 ± 0.08
Final biomass (g_DCW_.L^−1^)	10.1 ± 0.2	4.8 ± 0.1
Q_GLY_ (g.g_DCW_^−1^.h^−1^)	0.05 ± 0.01	—
Q_METH_ (g.g_DCW_^−1^.h^−1^)	—	0.02 ± 0.00
Q_SOR_(g.g_DCW_^−1^.h^−1^)	—	0.07 ± 0.01
Y_X/S_ (g_DCW_.molC^-1^)	16.8	8

Abbreviations: Q = carbon source consumption rate; GLY = glycerol; METH = methanol; SOR = sorbitol, Y_X/S_ = cell yield coefficient.

The probable reason behind the final *P. pastoris* RIY311 biomass being less than half of the maximum *Y. lipolytica* RIY368 biomass, despite consumption of more than 90% of carbon sources (Supplementary Fig. 2), is that some of the methanol was converted directly to CO_2_ via the dissimilatory pathway in *P. pastoris*, rather than to biomass via the assimilatory pathway. Similar findings were previously described^16^. This is further evidenced by the onset of stationary phase coinciding with sorbitol depletion, while more than 30% of methanol was still present at that time (Fig. 1 and Supplementary Fig. 2).

### CalB expression level

The expression level of the CalB gene was determined by qPCR in both yeast strains using the actin gene as a reference for normalization. Generally, it is assumed that DNA amplification occurred with the same efficiency in the different samples tested. However, this assumption could not be made here since actin gene sequence and primers used for amplification are different for *Y. lipolytica* strain RIY368 and *P. pastoris* strain RIY311. Therefore, the method of Pfaffl^24^, which takes into account this amplification efficiency, was used. As shown in Table 2, actin amplification occurred with different efficiencies in *Y. lipolytica* and *P. pastoris* (2.1 and 1.9, respectively). By contrast, CalB gene amplification occurred with the same efficiency as gene sequence and primers used were the same for both yeasts.

**Table 2:** Expression of the CalB-encoding gene in *Y. lipolytica* RIY368 and *P. pastoris* RIY311 during cultures in bioreactor. Sampling times correspond to the maximal gene expression observed. Error on measure was calculated by dividing the standard deviation by the mean value. Efficiency for actin amplification was determined according to the method of Pfaffl^23^. Measurements were performed in triplicate.

	*Y. lipolytica* RIY368	*P. pastoris* RIY311
Sampling time (h)	18	28
Mean value CT CalB	16.6	15.6
Error on measure CalB	0.06	0.07
Mean value CT actin	23.8	27.0
Error on measure actin	0.08	0.06
Efficiency actin	2.1	1.9
Pfaff fold difference	1	7

Abbreviation: CT = cycle threshold.

The CalB expression level was first determined for both yeast species grown in bioreactors at different time points (i.e., mid-exponential phase, end-of exponential phase and stationary phase) to highlight the time point when CalB gene expression was maximal. For *Y. lipolytica* strain RIY368, the maximal expression level was observed at the end of the exponential growth phase after glycerol depletion (i.e., after 18 h). In the extended culture period, CalB expression levels decreased drastically (around 30-fold). For *P. pastoris* strain RIY311, the maximal expression level was delayed to 28 h of growth, at which point more than 60% of methanol had been consumed (Supplementary Fig. 2). After that time period, methanol metabolism decelerated and lower levels of CalB expression were observed (around 8-fold). We then applied the mathematical model of Pfaffl for data obtained at maximal expression time point (i.e. after 18 and 28 h) to calculate the relative gene expression level between the two yeast species. As shown in Table 2, the relative CalB expression level was observed to be 7-fold higher in *P. pastoris* strain RIY311 than in *Y. lipolytica* strain RIY368.

### Lipase production in bioreactor

**Figure 2:**
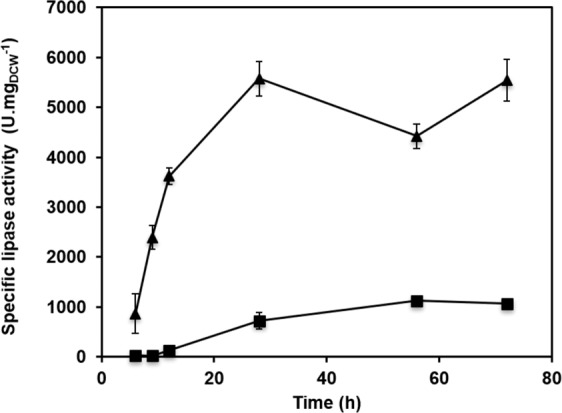
Lipase activities of *Y. lipolytica* RIY368 (triangles) and *P. pastoris* RIY311 (squares) during bioreactor cultivations. Cells were grown for 72 h in YSPGE and in YSPSM medium, respectively. Displayed values correspond to the means and standard deviations of independent duplicate experiments.

*Y. lipolytica* strain RIY368 and *P. pastoris* strain RIY311 were grown in bioreactors for 72 h in YSPGE medium and YSPSM medium, respectively. At various time points, lipase activity was measured in culture supernatant and specific activities were calculated. As shown in Fig. 2, specific lipase activities were significantly higher for *Y. lipolytica* strain RIY368. At 72 h, lipase activities of 5540 and 1066 U.mg_DCW_^−1^ were obtained for *Y. lipolytica* strain RIY368 and *P. pastoris* strain RIY311, respectively, representing thus more than a 5-fold difference. The kinetics of lipase production is also different for the two yeast species. For *Y. lipolytica* strain RIY368, lipase specific activity increased during all the exponential growth phase at a rate of 199 U.mg_DCW_^−1^.h^−1^, whereas for *P. pastoris* strain RIY311 it increased not only during the growth phase but also during a part of the stationary phase (up to 56 h) at a rate of 20 U.mg_DCW_^−1^.h^−1^. Regardless, these results highlight that the rate of extracellular lipase accumulation is almost 20-fold higher in *Y. lipolytica* strain RIY368 than in *P. pastoris* strain RIY311, and that this enzyme yield was produced within half the time period (i.e., 28 h vs 56 h).

### Elucidating the reasons behind the difference in lipase productivity

In order to determine the reasons behind the discrepancy between CalB expression level and enzyme productivity between *Y. lipolytica* and *P. pastoris*, we tested several hypotheses. Firstly, the presence of lipase inhibitory compounds in *P. pastoris* culture supernatant, such as methanol or metabolic by-products, was assessed. For that purpose, lipase samples from *Y. lipolytica* strain RIY368 culture supernatants were diluted in *P. pastoris* strain RIY311 culture supernatant (samples collected after 72 h of culture in bioreactor, see above section), rather than PBS used for the regular assays. After one hour of incubation at room temperature, the lipase activity was determined. Compared to control (i.e., sample diluted in PBS buffer), no significant reduction of lipase activity could be observed (18,197 ± 790 vs 20,057 ± 2596 U.ml^−1^; Supplementary Fig. 3). This highlighted that lipase activity in *P. pastoris* strain RIY311 culture supernatant was not inhibited, nor was the enzyme altered by residual methanol or metabolic by-products.

The second hypothesis tested was the incomplete protein maturation in *P. pastoris*, more precisely the incorrect cleavage of the pro-sequence, as CalB was cloned with its native pro-region in both yeast species in order to start with similar constructs. To test this hypothesis, CalB was expressed as a pro-enzyme in strain RIY311 and as a mature protein (i.e., without its native pro-sequence) in strain RIY314. In both strains, the alpha mating factor was used as a signal peptide. The two strains were grown in YSPSM medium for 20 h and the extracellular lipase activities were determined. As lipase specific activities were found of similar order in strains RIY311 and RIY314 (207 ± 32 vs 259 ± 13 U.mg_DCW_^−1^.L^−1^, Supplementary Fig. 4), it could be concluded that the CalB propeptide is not required for correct protein folding in *P. pastoris*, nor does it interfere with the catalytic activity of the enzyme through inefficient maturation. Therefore, inefficient maturation of the CalB protein was eliminated as the cause of the lower extracellular lipase activity observed for *P. pastoris* as compared to that of *Y. lipolytica*.

A third hypothesis to explain the low lipase activity in *P. pastoris* supernatants would be an intracellular accumulation of CalB protein. To test this hypothesis, strains expressing EGFP (RIY313, p*AOX1*-αMF-EGFP) and a fusion EGFP-CalB construct (RIY309, p*AOX1*-αMF-EGFP-CalB) were employed. In the CalB-EGFP fusion, the fluorescent protein was fused to the N-terminal part of CalB, based on the successful application of such a fusion for the lipase of *Rhizopus oryzae* (ROL)^25^. Strain RIY284 (p*AOX1*-EGFP) was used to illustrate the regular intracellular EGFP accumulation in the absence of secretion, while measurements of culture supernatants of strain RIY283 (MutS prototroph control strain) were used to define the fluorescence noise from the culture supernatants. Strains were grown in YSPSM medium, and the intracellular and extracellular fluorescence were monitored over time by means of flow cytometry and fluorometry, respectively, to test for intracellular *versus* extracellular EGFP and EGFP-CalB accumulation. CalB lipase activity was also determined in culture supernatant for strain RIY309. For both strains RIY313 (EGFP secreted) and RIY309 (EGFP-CalB secreted), EGFP intracellular fluorescence was 7-fold lower as compared to that of strain RIY284 (EGFP intracellular), and the fluorescence levels remained at the same average value (i.e., 7038 ± 1416 and 10,702 ± 2202 RFU, respectively; Supplementary Fig. 5) during the last 38 h of culture. Besides, a 287-fold increase in lipase activity of strain RIY309 over the cultivation time demonstrated accumulation of secreted EGFP-CalB rather than accumulation within the cells under those conditions (Supplementary Fig. 6). A similar observation was made for RIY313 (EGFP secreted), with a 7.5-fold increase in extracellular fluorescence (EGFP secreted), indicating again that the EGFP was secreted and accumulated over time in the culture supernatant (Supplementary Fig. 6). A potentially misleading positive effect of the EGFP fusion on CalB secretion was disregarded, as this type of fusion in fact led to inhibition of secretion in the case of ROL^26^.

Finally, it has been reported that the efficiency of *P. pastoris* to secrete recombinant proteins could be hampered by partial degradation along the secretion pathway. Detection of unfolded proteins in the endoplasmic reticulum (ER) triggers the unfolded protein response (UPR), which attenuates translation, increases protein folding capacity of the ER, and as a last resort alleviates cellular stress via ER-associated protein degradation (ERAD)^27^. Misfolded proteins, for their part, are straightforwardly directed to ERAD. The latter involves retro-translocation to the cytosol, ubiquitinylation and proteasomal degradation^28,29^. Pfeffer *et al*.^29^ reported for complex proteins such as antibody fragment that up to 60% of the newly synthesized protein was degraded intracellularly.

**Figure 3:**
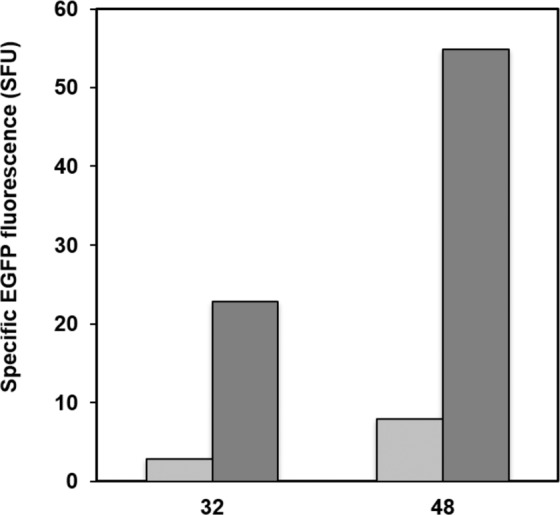
Specific EGFP extracellular fluorescence of strain RIY313 (p*AOX1*-αMF-GFP) during culture in presence (dark grey) or absence (light grey) of proteasome inhibitor MG-132. Fluorescence was determined by spectrophotometry after 32 h and 48 h of culture in YSPSM medium. Fluorescence values were normalized to biomass values at the corresponding time. Displayed values correspond to the means of independent triplicate experiments. Standard deviations were less than 19.0% in presence and 5.3% in absence of MG-132, respectively.

MG-132 is a well-known proteasome inhibitor that inhibits the activity of serine and cysteine proteases^30^. It has successfully been used to increase secreted protein titer^31^. We first tested the effect of MG-132 on CalB secretion but the results were unexpected, as the lipase activity obtained in the culture containing the proteasome inhibitor were up to 10-fold lower than a culture without MG-132. As a potential explanation, we hypothesized that MG-132 inhibits the enzymatic activity of lipases, since they are serine-active enzymes. As an alternative, we tested the effect of the proteasome inhibitor on secreted EGFP (strain RIY313). As shown in Fig. 3, the specific fluorescence was on average 2.5-fold increased in the presence of the proteasome inhibitor (3 vs 8 FU.g_DCW_^−1^ after 32 h, and 55 vs 23 FU.g_DCW_^−1^ after 48 h). This highlighted that some CalB was lost due to intracellular degradation either in the cytosol^32^ or in vacuoles^33,34^ prior to secretion, thus explaining the low lipase titer observed in the culture supernatant of *P. pastoris* strain RIY311, despite higher expression levels of CalB than in *Y. lipolytica* strain RIY368.

**Figure 4:**
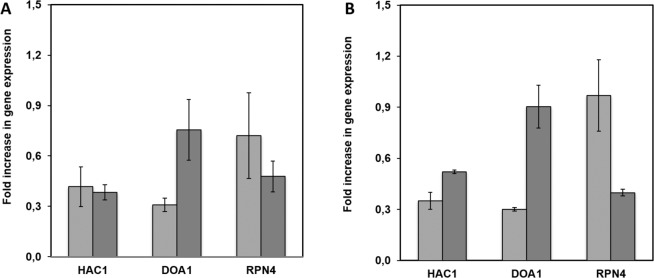
Relative expression level of genes *HAC1*, *DOA1* and *RPN4* in strains RIY311 (p*AOX1*-CalB, panel A) and RIY313 (p*AOX1*-αMF-EGFP, panel B). Expression levels were normalised according to that of actin and compared to that of strain RIY283 (MutS). Samples were collected after 24 h (light grey) and 48 h (dark grey) of growth in YSPSM medium. Displayed values correspond to means and standard deviations of independent triplicate experiments.

To further confirm this hypothesis, the expression levels of genes involved in UPR, ERAD or proteasomal degradation process were monitored after 24 h and 48 h of culture of strains RIY311 (p*AOX1*-pro-CalB) and RIY283 (MutS control strain) in YSPSM medium. The selected genes were *HAC1* (an unfolded protein response transcription factor^35^), *DOA1* (involved in the ubiquitin-dependent protein degradation^36^) and *RPN4* (a transcription factor of proteasomal gene^37^). As shown in Fig. 4, the expression levels of the three tested genes were found significantly higher after 24 h and 48 h of culture in strain RIY311, as compared to control strain RIY283, thus confirming that ERAD is responsible for the loss of CalB through proteasome degradation. Similar observations were also made with strain RIY313 that secretes EGFP. Therefore, the most likely explanation for the lower extracellular CalB activity in *P. pastoris* strain RIY311 than in *Y. lipolytica* strain RIY368, despite the opposite trend in terms of expression level of CalB-encoding gene, is that CalB is degraded before its secretion by the proteasome of *P. pastoris* strain RIY311. The high expression levels afforded by the MutS phenotype leads to an overloading of the ER machinery. This could involve either insufficient folding capacity within the ER and hence translocation to the cytosol through ERAD; or insufficient translocation capacity to enter the ER in the first place, as suggested by Zahrl and co-workers^32^. The obtained transcriptional data however proves that the ERAD pathway is up-regulated and is hence capable of at least contributing to the determined proteasomal degradation.

The theory that the obtained results are due to abnormally high recombinant protein levels challenging the processing capacity of the ER, is in agreement with previous results, in which the difference in intracellular EGFP levels between Mut+ and MutS was greater than the difference in extracellular EGFP levels, indicating that although more recombinant proteins were produced, secretion remained limiting^16^.

To put the general findings obtained during this study into context, it is important to bear in mind that while *P. pastoris* is naturally capable of producing large amounts of proteins in response to methanol (which is indeed the basis of the popular methanol-induced AOX promoter system), it is not naturally prone to high-level secretion of proteins. *Y. lipolytica*, by contrast, naturally secretes high levels of various native proteins, including proteases, RNases and lipases (reviewed by Barth and Gaillardin^38^).

## Conclusion

The purpose of this study was to directly compare the efficiency of *Y. lipolytica* and *P. pastoris* to synthetize and secrete rProt in bioreactor conditions, as such a direct comparison has not been reported so far. For this purpose, the lipase CalB was used as a model protein since it is an enzyme of industrial interest. *Y. lipolytica* was found to perform better in bioreactor cultures, in terms of cell growth, enzyme titer, and production time. This was despite *P. pastoris* yielding significantly higher levels of CalB transcript compared to *Y. lipolytica*.

Various possible explanations for the observed inconsistency between specific mRNA levels and final protein yields were investigated, and the most likely explanation was found to be that a proportion of the rProt is degraded before it can be secreted.

These results have highlighted the multi-faceted nature of recombinant protein production and secretion. It was demonstrated that superior expression levels may not necessarily lead to higher rProt yields, as other factors involved in the global process also need to be considered.

Based on the results of the present study, the described *Y. lipolytica* system appears to be superior for recombinant lipase production and secretion to the more commonly used *P. pastoris* system.

## Methods

### Growth and culture conditions

*Escherichia coli* strains were grown at 37 °C in Luria-Bertani medium supplemented with kanamycin sulfate (50 mg.L^−1^). *P. pastoris* and *Y. lipolytica* strains were grown at 30 °C in YPD medium or in buffered rich medium supplemented with appropriate carbon sources for each yeast. The buffered rich medium consisted in 6.7 g.L^−1^ YNB without amino acids, potassium phosphate buffer 100 mM pH 6, 10 g.L^−1^ yeast extract, 20 g.L^−1^ soytone, 0.4 mg.L^−1^ biotin, and 1.2% PTM1 solution. For *Y. lipolytica*, the buffered rich medium was supplemented with 20 g.L^−1^ glycerol and 10 g.L^−1^ erythritol as carbon source and inducer, respectively (YSPGE). For *P. pastoris*, the buffered rich medium was supplemented with 9.44 g.L^−1^ sorbitol and 10 g.L^−1^ methanol as carbon source and carbon source / inducer, respectively (YSPSM). Yeast cultures were inoculated at an initial optical density at 600 nm (OD_600_) of 0.5. MG-132 was used at a final concentration of 500 μM (stock solution at 50 mM in DMSO). Shake-flask cultures were performed for 48 h in triplicate in 100- or 250-mL flasks, with incubation at 30 °C and 150 rpm. Bioreactor cultures were performed for 72 h in duplicate in DASGIP DASbox Mini Bioreactors SR0250ODLS (Eppendorf, Hamburg, Germany). Temperature was set at 30 °C, agitation at 800 rpm and aeration at 1 vvm. pH was automatically adjusted to 6 by addition of 42.5% H_3_PO_4_ (8 M) or NaOH (12.5 M).

### General genetic techniques

Standard media and techniques were used for *E. coli*^33^, for *P. pastoris*^39^ and for *Y. lipolytica*^5^. *P. pastoris* was transformed according to the method described by Lin-Cereghino *et al*.^40^, and *Y. lipolytica* according the lithium acetate method^41^. Restriction enzymes, DNA polymerases and T4 DNA ligase were obtained from New England Biolabs (NEB, Ipswich, MA, USA). Primers for PCR amplifications were synthetized by Eurogentec (Seraing, Belgium, Supplementary Table 1). DNA fragment were purified from agarose gels using Monarch DNA purification kit (New England Biolabs). DNA sequencing was performed by GATC Biotech (Konstanz, Germany). Transcriptional analyses were performed by quantitative PCR (qPCR) with primers described in Supplementary Table 1, using the actin gene as reference. Total RNA was extracted using the NucleoSpin RNA Plus kit (Machery-Nagel, Düren, Germany) according to the manufacturer’s recommendations. Total cDNA was prepared by reverse transcription using the Reverse Transcriptase Core kit (Eurogentec) according to the manufacturer’s recommendations. qPCR was performed using the Luna Universal qPCR Master Mix and the StepOnePlus Real-Time PCR system (ThermoScientific, Waltham, MA, USA). Data were analyzed using StepOne software v2.3. Codon content of CalB gene sequence was analyzed for less abundant and rare codons (below 20% and 10% in frequency in the host strain, respectively) using Graphical Codon Analyzer CGUA (http://gcua.schoedl.de). Codon usage tables were retrieved from http://www.kazusa.or.jp.

### Plasmids and strains construction

Strains and plasmids used in this study are listed in Supplementary Table 2. Construction of *Y. lipolytica* strain RIY368 is described in Park *et al*.^11^. Briefly, the DNA fragment containing the CalB sequence codon optimized for *Y. lipolytica* by Biocatalysts LTD (Cardiff, UK) and synthesized by Geneart (Regensburg, Germany) was cloned as a *BamH1*/*AvrII* fragment in vector JME4266 at the corresponding restriction sites. The *pEYK1–3AB*-CalB expression cassette was then obtained by *Not*I digestion and used to transform strain JMY7126. The resulting strain JMY7539 was transformed with vector RIE279 (zeta-*LYS5*-zeta) to render the strain prototroph. The resulting strain was designated RIY368.

For *P. pastoris* vector construction, the gene encoding lipase (CalB) was PCR amplified from the vector JMP6266 using the primer CalB-R together with either CalBPro-F to include the pro-region, or CalBMat-F for the mature protein sequence. The resulting amplicons were cloned into expression vector pIB4 using the *Eco*RI and *Not*I restriction sites, yielding the vectors RIP254 (pro-peptide) and RIP255 (mature peptide). The full-length *Saccharomyces cerevisiae* secretion signal for α-mating factor (αMF) was amplified from plasmid pPTK005–3a-αMF (RIP252) using the primers αMF-F and αMF-R. The resulting ~270 bp fragment was digested with *EcoR*I and cloned in RIP254 and RIP255 at the corresponding sites, yielding vectors RIP256 (pro-peptide) and RIP257 (mature peptide), respectively. The sequence encoding the mature CalB peptide was also cloned into plasmid RIP233 (p*AOX1*- αMF-EGFP)^16^ using *Not*I, resulting in plasmid RIP259 (p*AOX1*- αMF-EGFP-CalB).

These vectors were then digested with *Stu*I prior to transformation of *P. pastoris* strain RIY282 (MutS)^16^ to yield strains RIY311 (CalB pro-peptide), RIY314 (CalB mature peptide) and RIY309 (EGFP fused to CalB mature peptide). Construction of strains RIY284 and RIY313 were described in Theron *et al*.^16^.

### Analytical methods

Cell growth was monitored either by optical density at 600 nm (OD_600_) or dry cell weight (DCW) as previously described^18^. Methanol, sorbitol, glycerol, erythritol and erythrulose concentrations were determined by isocratic RID-HPLC (Agilent 1100 series equipped with UV and RID detector, Agilent Technologies, Santa Clara, CA, USA) using an Aminex HPX-87H ion-exclusion column (300 × 7.8 mm Bio-Rad, Hercules, CA, USA) with 5 mM H_2_SO_4_ as mobile phase at a flow rate of 0.5 mL.min^−1^ at 65 °C.

Cell fluorescence was quantified using a BD Accuri C6 Flow Cytometer (BD Biosciences, San Jose, CA, USA). Excitation was performed with a 20-mW, 488-nm solid-state blue laser, the emission wavelength was measured at 533 nm. For each sample, 20,000 cells were analyzed using the FL1 channel to identify the fluorescence associated with the synthesis of the enhanced green fluorescent protein (EGFP), while FSC channel served to estimate the diameter of analyzed cells. For both the FL1 and FSC, the area of the global signal was recorded (FL1-A, FSC-A). The flow cytometry dot-plots (FL1/FSC) were analyzed using CFlowPlus software (Accuri, BD Biosciences). EGFP fluorescence was defined as the median fluorescence value of a considered sample minus endogenous florescence value (from parent strain RIY283) at the corresponding time. It was expressed as relative fluorescence units (RFU).

Spectrophotometric analysis of EGFP in culture supernatants was performed on SpectraMax M2 (Molecular Devices, San Jose, CA, USA) using λ_ex_ and λ_em_ at 488 and 535 nm, respectively. Measurements were taken after 30 s of sample shaking. Signal gain was set to 225, and the number of light flashes was set to 30. Specific EGFP fluorescence was expressed as specific fluorescence units (SFU), i.e., as fluorescence value normalized to biomass and from which endogenous fluorescence was deduced.

The lipase activity in the culture supernatant was determined by monitoring the hydrolysis of *p*-nitrophenyl butyrate (p-NPB) to *p*-nitrophenol (p-NP) at 405 nm (A_405_), as described by Fickers *et al*.^42^, using a UVIKON XL spectrophotometer (BioTek Instruments, Winooski, VT, USA). Supernatant samples were diluted to obtain initial velocities below A_405_ of 0.3 U.min^−1^. All lipase activity assays were performed at least in duplicate on two independent cultures. One unit of lipase activity was defined as the amount of enzyme releasing 1 µmol *p*-nitrophenol per minute at 25 °C and pH 7.2 (εPNP = 0.0148 µM^−1^.cm^−1^).

## Supplementary information

Supplementary information.

## Data Availability

All relevant data are provided in the manuscript and the Supporting Information files.
