# Supplementary material for: Comprehensive comparison of Yarrowia lipolytica and Pichia pastoris for production of Candida antarctica lipase B
Source: Sci Rep. 2020 Feb 3;10:1741. doi: 10.1038/s41598-020-58683-3 (PMC6997362; doi:10.1038/s41598-020-58683-3)

Comprehensive comparison of *Yarrowia lipolytica* and *Pichia pastoris* for production of *Candida antarctica* lipase B

Chrispian W. Theron[1,*], Marie Vandermies[1,*], Samuel Telek[1], Sebastien Steels[1], Patrick Fickers[1]

[*] Contributed equally to this work

[1]Microbial Processes and Interactions, TERRA Teaching and Research Centre, University of Liège - Gembloux AgroBio Tech, Avenue de la Faculté, 2. B-5030 Gembloux, Belgium; Tel: +32 81 622 311.

Corresponding author:
Patrick Fickers
Microbial Processes and Interactions, TERRA Teaching and Research Centre, University of Liège - Gembloux AgroBio Tech, Avenue de la Faculté, 2. B-5030 Gembloux, Belgium; Tel: +32 81 622 311.

**Supplementary Table 1: Primers used in this study**		
**Primer**	**Sequence (5'-3')**	**Restriction site***
YLact-F-qPCR	TCCAGGCCGTCCTCTCCC	
YLact-R-qPCR	GGCCAGCCATATCGAGTCGCA	
PPact-F-qPCR	AGATGGCTCCGAGAAGTTCA	
PPact-R-qPCR	GTTGCTCAGAGGGCTTCAAC	
CalB-F-qPCR	TCTCTGCTCCTTCTGTGTGG	
CalB-R-qPCR	GTCGAACAGAGGTCCACAGA	
CalBPro-F	**GCGGCCGC**T**GAATTC**ATGACCCCTCTGGTGAAGCGACTG	*Not*I, *EcoR*I
CalBMat-F	**GCGGCCGC**T**GAATTC**ATGCTGCCTTCTGGATCTGACCCTGC	*Not*I, *EcoR*I
CalB-R	**GGATCC**TTAA**GCGGCCGC**AGGGGTGACAATACCAGAACAGG	*BamH*I, *Not*I
αMF-F	GACTG**GAATTC**ATGAGATTTCCTTCAATTTTTACTGCTG	*EcoR*I
αMF-R	**GAATTC**AGCTTCAGCCTCTCTTTTCTCGAG	*EcoR*I

* Restriction sites are indicated in bold in the table.

Supplementary Table 2: Strains and plasmids used in this study

Strain (plasmid)	Genotype	Reference
E. coli		
JMP4266 (JME4266)	JMP62-*URA3ex*-p*EYK1-3AB*	[1]
RIE158 (RIP158)	pIB4 (p*AOX1-HIS4*)	Addgene plasmid #25453
JME4365 (JMP4365)	JMP62-*URA3ex*-p*EYK1-3AB*-CalB	[1]
RIE233 (RIP233)	p*AOX1*-αMF-EGFP-*HIS4*	This work
RIE279 (RIP279)	Zeta-LYS5ex-Zeta	This work
RIE252 (RIP252)	pPTK005-3a-αMF	Addgene PTK005-3a-Amf
RIE254 (RIP254)	p*AOX1*-pro-CalB-*HIS4*	This work
RIE255 (RIP255)	p*AOX1*-CalB (mature)-*HIS4*	This work
RIE256 (RIP256)	p*AOX1*-αMF-pro-CalB-*HIS4*	This work
RIE257 (RIP257)	p*AOX1*-αMF-CalB (mature)-*HIS4*	This work
RIE259 (RIP259)	p*AOX1*-αMF-EGFP-CalB (mature)-*HIS4*	This work
Y. lipolytica		
RIY368 (JMY7539)	*MatA, ura3-302, leu2-270-LEU2-ZETA, xpr2-322, lip2Δ, lip7Δ, lip8Δ, lys5Δ, eyk1Δ, pEYK1-3AB*-CalB-*URA3ex, LYS5ex*	[1]
P. pastoris		
RIY282	GS115 (*his4*, MutS; *AOX1::bleR*)	[2]
RIY283	RIY282, *HIS4* prototrophy restored	[2]
RIY284	RIY282 + p*AOX1*-EGFP	[2]
RIY309	RIY282 + p*AOX1*-αMF-EGFP-CalB (mature)	[2]
RIY311	RIY282 + p*AOX1*-αMF-pro-CalB	This work
RIY313	RIY282 + p*AOX1*-αMF-EGFP	[2]
RIY314	RIY282 + p*AOX1*-αMF-CalB (mature)	This work

1. Park, Y.-K. *et al.* Efficient expression vectors and host strain for the production of recombinant proteins by *Yarrowia lipolytica* in process conditions. *Microb. Cell Fact.* **18**,167(2019).

2. Theron, C. W. *et al.* Expression of recombinant enhanced green fluorescent protein provides insight into foreign gene expression differences between MUT+ and MUTS strains of *Pichia pastoris*. *Yeast* **36**, 285–296 (2019).

Figure S1: Nucleotide sequence of pro-CalB. Font code is the following: bold font, pro sequence of CalB; normal font, mature sequence of CalB; underlined font, codon with frequency less than 20% in *Y. lipolytica.* The number underneath the underlined codons indicate their frequency in *Y. lipolytica* codon usage table.

ACC CCT CTG GTG AAG CGA CTG CCT TCT GGA TCT GAC CCT GCC TTC TCT CAG CCC AAG TCT GTT
CTG GAC GCT GGT CTG ACC TGT CAG GGA GCT TCT CCT TCT TCT GTG TCT AAG CCC ATT CTC CTG
GTG CCT GGA ACC GGA ACC ACC GGT CCT CAG TCT TTC GAC TCG AAC TGG ATT CCT CTG TCT ACC
16
CAG CTG GGA TAC ACC CCC TGT TGG ATT TCT CCT CCT CCT TTC ATG CTG AAC GAC ACC CAG GTG
AAC ACC GAG TAC ATG GTG AAC GCC ATT ACC GCT CTG TAC GCT GGC TCT GGA AAC AAC AAG CTG
CCC GTT CTG ACC TGG TCT CAG GGA GGT CTG GTG GCT CAG TGG GGT CTG ACC TTC TTC CCT TCT
ATT CGA TCT AAG GTG GAC CGA CTG ATG GCC TTC GCT CCC GAC TAC AAG GGA ACC GTT CTG GCT
GGT CCT CTG GAC GCT CTG GCT GTC TCT GCT CCT TCT GTG TGG CAG CAG ACC ACC GGC TCT GCT
CTG ACC ACC GCT CTG CGA AAC GCT GGA GGT CTG ACC CAG ATT GTC CCC ACC ACC AAC CTG TAC
TCT GCC ACC GAC GAG ATT GTC CAG CCT CAG GTG TCT AAC TCT CCT CTG GAC TCT TCG TAC CTG
16
TTC AAC GGA AAG AAC ATT CAG GCT CAG GCT GTC TGT GGA CCT CTG TTC GAC ATT GAC CAC GCT
GGC TCT CTG ACC TCT CAG TTC TCC TAC GTG GTT GGA CGA TCT GCT CTG CGA TCT ACC ACC GGT
CAG GCT CGA TCT GCT GAC TAC GGT ATC ACC GAC TGT AAC CCT CTG CCT GCC AAC GAC CTG ACC
CCT GAG CAG AAG GTG GCT GCT GCT GCT CTG CTG GCT CCC GAG GCT GCT GCC ATT GTC GCT GGT
CCC AAG CAG AAC TGC GAG CCC GAC CTG ATG CCT TAC GCT CGA CCC TTC GCT GTT GGA AAG CGA
ACC TGT TCT GGT ATT GTC ACC CCT TAA

Figure S2 : Substrate uptake during bioreactor cultures of *Y. lipolytica* strain RIY368 (Panel A; triangles, erythritol; circles, glycerol) and *P. pastoris* strain RIY311 (Panel B; triangles, methanol; circles, sorbitol). Cells were grown for 72 h in YSPGE and in YSPSM medium, respectively. Displayed values correspond to the means and standard deviations of independent duplicate experiments.

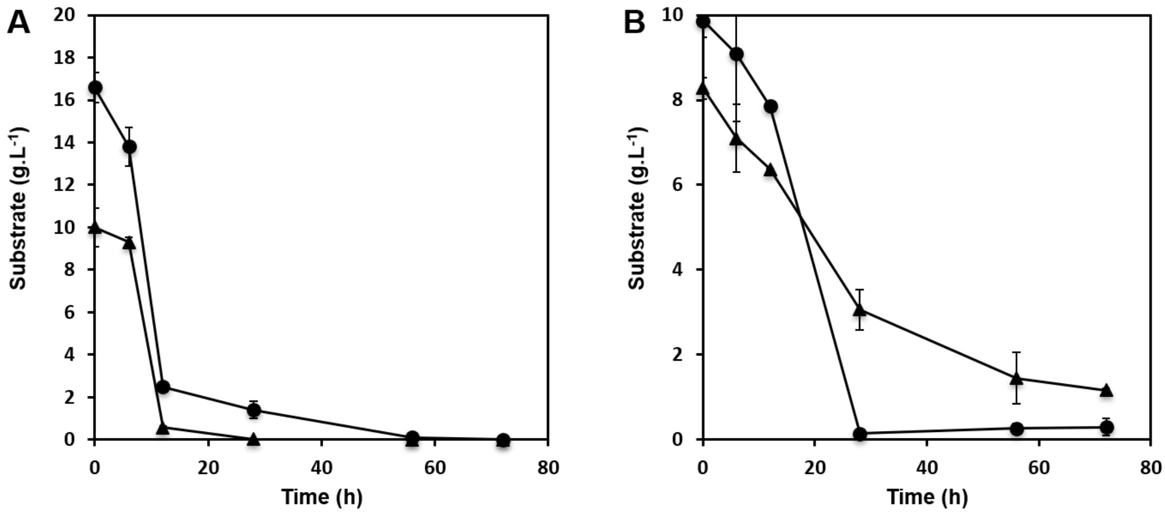

Figure S3: Lipase activity of culture supernatant of *Y. lipolytica* strain RIY368 diluted 40 times in PBS buffer and in *P. pastoris* strain RIY311 supernatant (samples collected after 72 h). Activity was measured after one hour of incubation at room temperature. Displayed values correspond to the means and standard deviations of independent triplicate experiments.

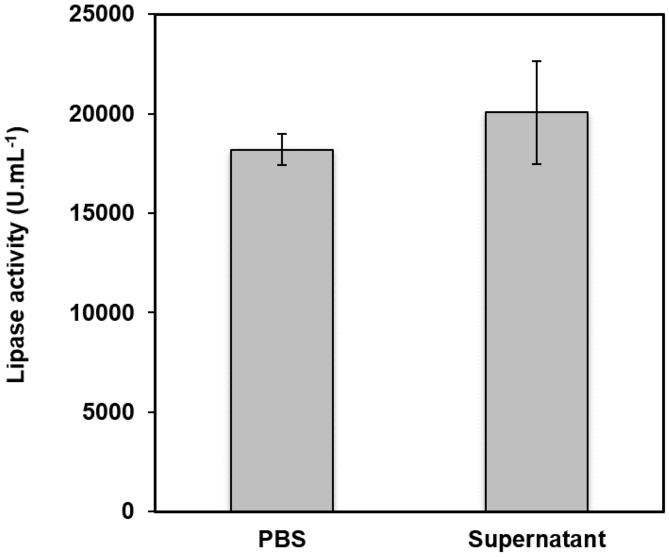

Figure S4: Specific lipase activity in culture supernatants of strains RIY311 (pro-CalB) and RIY314 (mature CalB). Samples were collected after 20 h of growth in buffered rich medium. Displayed values correspond to the means and standard deviations of independent triplicate experiments.

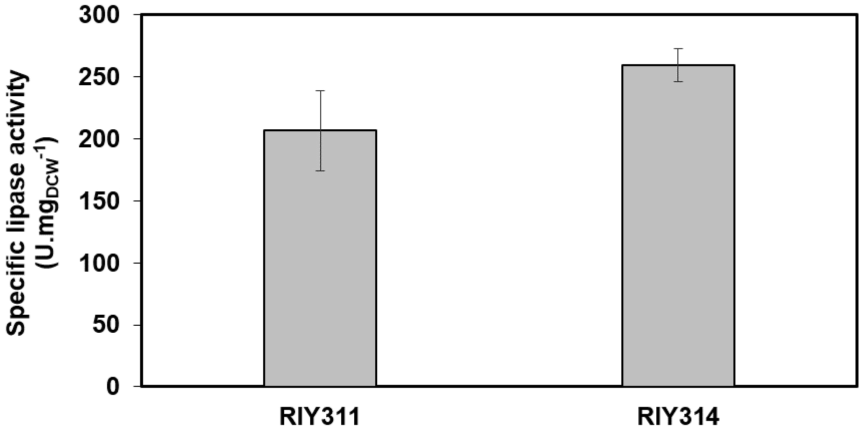

Figure S5: EGFP intracellular fluorescence of strains RIY284 (p*AOX1*-EGFP, triangles), RIY313 (p*AOX1*-αMF-EGFP, squares) and RIY309 (p*AOX1*-αMF-EGFP-CalB, circles). Fluorescence was determined by flow cytometry and endogenous florescence values (from parent strain RIY283) were deduced from the raw fluorescence of the different stains. Displayed values correspond to the means of independent triplicate experiments. Standard deviations were less than 8.6%.

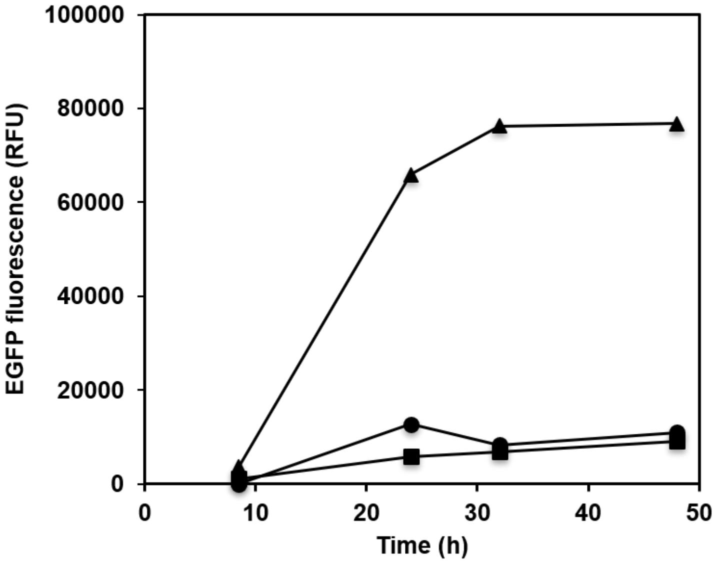

Figure S6: Specific EGFP extracellular fluorescence of strain RIY313 (p*AOX1*-αMF-EGFP, squares) and specific lipase activity of strain RIY309 (p*AOX1*-αMF-EGFP-CalB, circles). Raw fluorescence values were determined by spectrophotometry, normalized to biomass, and endogenous florescence values (from parent strain RIY283) were deduced in order to obtain the final fluorescence values. Displayed values correspond to the means of independent triplicate experiments. Standard deviations were less than 4.2%.

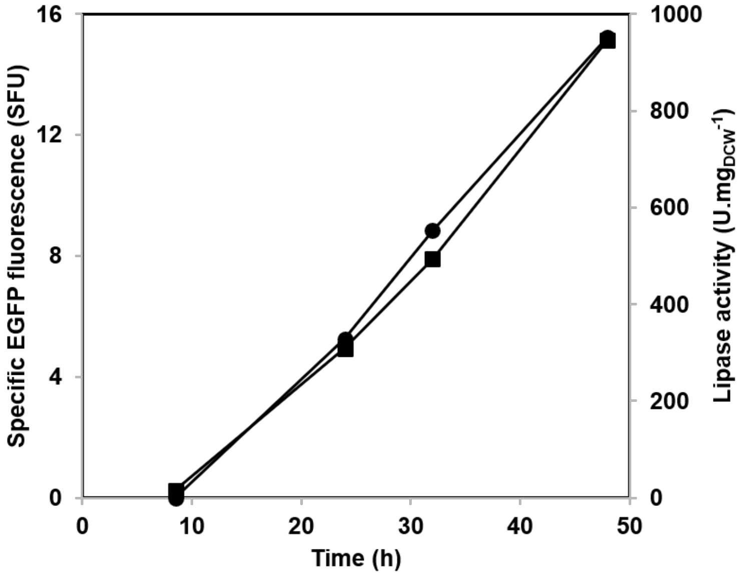

Supplement: Supplementary file 1 — Supplementary information. [file 41598_2020_58683_MOESM1_ESM.pdf]
